# Association of three different dietary patterns with the metabolomic profiles of children: the BiomarKid project

**DOI:** 10.1007/s00394-026-04098-1

**Published:** 2026-09-12

**Authors:** Irina Gheorghita, Lara Vehovec, Veit Grote, Jeannie Horak, Mariona Gispert-Llauradó, Natalia Ferré, Berthold Koletzko, Hans Demmelmair, Klaus Kratochwill, Elvira Verduci, Dariusz Gruszfeld, Joaquin Escribano, Gabi Kastenmüller, Veronica Luque

**Affiliations:** 1 Pediatric Nutrition and Human Development Research Unit (PediNuR), Universitat Rovira i Virgili (URV), C/ Sant Llorenç 21, 43201 Reus, Spain; 2 Institut de Recerca Biomèdica Catalunya Sud (IRBCatSud), Reus, Spain; 3 Helmholtz Zentrum München – German Research Center for Environmental Health, Institute of Computational Biology, Ingolstädter Landstraße 1, D-85764 Neuherberg, Munich, Germany; 4 Department of Paediatrics, Dr. von Hauner Children’s Hospital, LMU Hospital, LMU-Ludwig-Maximilians-Universität Munich, Munich, Germany; 5 German Center for Child and Adolescent Health, Munich, Germany; 6 Dept. Pediatrics, Chulalongkorn University, Bangkok, Thailand; 7 Core Facility Proteomics, Medical University of Vienna, Vienna, Austria; 8 Division of Pediatric Nephrology and Gastroenterology, Department of Pediatrics and Adolescent Medicine, Comprehensive Center for Pediatrics, Medical University of Vienna, Vienna, Austria; 9 Department of Health Sciences, University of Milan, Milan, Italy; 10 Pediatric Unit, Foundation IRCCS Ca’ Granda Ospedale Maggiore Policlinico, Milan, Italy; 11 Neonatal Department, Children’s Memorial Health Institute, Warsaw, Poland; 12 Hospital Universitari Sant Joan de Reus, Reus, Spain; 13 Serra Hunter Fellowship, Generalitat de Catalunya, Barcelona, Spain

**Keywords:** Children, Dietary patterns, Food groups, Metabolomics, Metabolite signatures

## Abstract

**Background and aims:**

Metabolomics offers a promising approach to objectively assess food intake and nutrition-related health risks. Three different dietary patterns (DPs) associated with cardiometabolic health were previously identified at 2 years of age in the EU Childhood Obesity Project and were used as reference patterns for longitudinal scoring throughout childhood: “Core foods” DP (CORE), rich in vegetables, fruits, fish, meat, and olive oil; “Animal protein sources” DP (PROT), high in white meat, processed meat, flavoured milk, snacks, fish, and vegetables; and “Poor-quality fats and sugars” DP (F&S), high in soft cheese, butter, added sugars, and juices. This study aimed to identify metabolomic signatures of these DPs.

**Methods:**

Targeted liquid chromatography-tandem mass spectrometry quantified 236 metabolites in blood samples collected at ages 5.5 and 8 years from 421 children, with 156 children contributing samples at both timepoints. Associations between DPs and metabolite levels were assessed using adjusted linear mixed models. Metabolite set enrichment analysis (MSEA) was conducted across 23 distinct sets of related metabolites.

**Results:**

Forty-eight metabolites associated with DPs, although they did not remain significant after false discovery rate correction. Five showed associations with multiple DPs. For example, LPC a C22:6 showed associations with opposite effect directions for CORE and F&S (β = 0.108, *p* = 0.038, q = 0.539; β=− 0.140, *p* = 0.025, q = 0.484, respectively). The ratio PC aa C40:5/PC aa C40:6 was negatively associated with CORE (β=− 0.207, *p* = 3.81 × 10^− 5^, q = 0.016) and positively with F&S (β = 0.136, *p* = 0.020, q = 0.463). CORE and F&S showed opposite directions in seven of the eight metabolite groups significant in the MSEA. A group of phosphatidylcholines containing polyunsaturated fatty acids (including DHA) was positively associated with PROT (NES: 1.837, *p* = 0.001, q = 0.017).

**Conclusions:**

Distinct metabolite groups were associated with adherence to specific dietary patterns in children. Consistent findings across metabolite classes and dietary components suggest biologically plausible metabolomic signatures that require confirmation in independent cohorts.

**Supplementary Information:**

The online version contains supplementary material available at https://doi.org/10.1007/s00394-026-04098-1.

## Introduction

Diet is an important contributor to health promotion and disease prevention [1]. However, accurately assessing dietary intake remains a major challenge in nutritional epidemiology [2]. Individuals often do not reliably report their dietary intake, and this challenge becomes even greater in children, where information is frequently obtained from proxy reporters (e.g., family members or day-care attendants), leading to over-reporting and under-reporting rates estimated at around 19% and 10.6%, respectively [2].

Although improving dietary assessment is important, accurately characterising the relationship between diet and metabolism also requires accounting for differences in how individuals metabolically respond to dietary exposures [3, 4]. This inter-individual variability in metabolic responses is influenced by a variety of factors, including genetics, age, biological variables (such as absorption, excretion) [5], environmental conditions, and other lifestyle factors [6], making it challenging to objectively estimate the association between diet and their metabolic outcomes [7].

Metabolomics has emerged as a powerful approach for the comprehensive characterization of metabolic pathways, constituting a complex network of interrelated biochemical reactions [8]. By simultaneously profiling a wide range of metabolites, metabolomics enables the identification of metabolic profiles associated with environmental exposures, including diet, and with health and disease outcomes [4, 6, 9]. Metabolomics applied to nutritional research, known as nutrimetabolomics, provides a powerful tool for identifying metabolite signatures of dietary habits and for elucidating the complex biological pathways through which diet influences metabolism and long-term health [10, 11].

Available literature about nutrimetabolomics mainly focused on specific food groups rather than the overall diet, particularly in paediatric cohorts [12]. For example, hippurate was reported as a robust biomarker for fruit and vegetables consumption in children [13]. However, diet influences metabolism through complex interactions between its multiple components. Thus, analysing dietary patterns (DPs) is essential to capture these combined effects of diet on health [14–17].

The present study aimed to identify individual metabolites and groups of metabolites associated with DPs in European children.

## Materials and methods

### Study design and population

Data were obtained from the EU Childhood Obesity Project (EU CHOP) trial, initially designed as a double-blind, randomized clinical trial conducted in healthy infants from Germany, Belgium, Italy, Poland, and Spain during the first year of life (October 2002–July 2005) [18]. The study included an observational follow-up with multiple visits until 8 years of age. Detailed descriptions of the EU CHOP study are available elsewhere [18–20]. The EU CHOP consortium later contributed to the BiomarKid project (Biomarker signatures of diet, physical activity and sleep in children and youth; https://www.healthydietforhealthylife.eu/project/biomarkid). The main objective of BiomarKid was to characterize metabolomic and proteomic signatures of dietary patterns, physical activity, sedentary behaviour, and sleep and their interactions using omics approaches. For the present analysis, we used data from visits at ages ~ 5.5 and ~ 8 years, based on the availability of both dietary and metabolomic data at each timepoint. The analyses were conducted cross-sectionally using the two timepoints.

### Dietary data

Three-day food records were used to capture dietary intake of children, completed by caregivers at several timepoints from ages 2 to 8 years. Information on procedures for assessing dietary intake was published [21].

For the DP analysis, the food items were classified based on their nutritional profile and level of food processing into 29 major food groups [18]. An exploratory factor analysis was applied to dietary data at 2 years, and three different DP were identified. The first one was labelled as “Core foods” DP (CORE), characterized by high consumption of vegetables, fruits, fish, olive oil, white and red meat. The second was named “Animal protein sources” DP (PROT), as it was positively loaded by white and processed meat, fish, eggs, flavoured milk, vegetables and snacks. The third was labelled “Poor-quality fats and sugars” DP (F&S), as it was characterized by high consumption of saturated spreads, such as butter, added sugars, and fruit juices and soft cheese, and negatively loaded for olive oil and fish. The three DPs were extracted at several timepoints until 8 years and were consistently identified across all timepoints. There were minor differences in factor loadings and the contribution of specific food groups across ages, but the overall structure and interpretation of these three DPs were remarkably stable throughout childhood [18]. To enable longitudinal assessment of adherence to the same dietary patterns across childhood, pattern coefficients derived at 2 years were applied at subsequent timepoints. This approach was chosen to ensure comparability of dietary pattern scores over time, as age-specific patterns would not be directly comparable across repeated assessments. Additional information on factor loadings can be found elsewhere [15]. Each participant received an adherence score for each DP at the respective timepoints (5 and 8 years).

The dietary intake was converted into nutrients (carbohydrates, sugars, fats, proteins, fibre) using a Nutrcalc software [21], based on BLS II.3 food composition [22]. In addition, protein was categorized as animal (other than dairy), dairy and plant protein; fats were categorized as healthy fats (from olive oil, nuts, and fish) or unhealthy fats (sum of processed and animal fats). Processed fats were those from bakery products, butter, and pastries; animal fats were those from meat, eggs, and dairy (Supplementary Material 1).

### Metabolite profiling

Blood samples were collected in ethylenediaminetetraacetic acid (EDTA) tubes at the ages of 5.5 and 8 years, centrifuged and stored at study centres at − 70 °C and − 80 °C, respectively. Fasting of at least 6 h was intended but was not always possible, particularly in Germany, and the participant’s fasting status was recorded. Plasma metabolites were quantified at the Dr. von Hauner Kinderspital (Ludwig-Maximilians-University, Munich at the Department of Paediatrics, Children’s Hospital), using targeted mass-spectrometry (MS)-based metabolomics. A detailed description of biological sample preparation, instruments and analysis protocols used was published by Kirchberg et al. [23], and information about annotation, labelling and quantification was provided in Supplementary Material 2.

Samples from ages 5.5 and 8 years were measured separately at their times of availability. Measurements were performed in six batches per each platform and timepoint. Each batch contained at least six quality control (QC) samples and four samples of human control plasma (CP) at two different dilutions (CP-I and CP-II) from RECIPE (Munich, Germany). Exception was flow-injection analysis–mass spectrometry (FIA–MS/MS) platform, where CP samples were not measured. Metabolomics data QC was performed as detailed in the Supplementary material. After QC, 272 and 258 metabolites remained at 5.5 and 8 years, respectively. Missing values were imputed separately for the two timepoints employing R-package *missForest* (version 1.5) [24], which uses a random forest approach. For analyses in this study, the 236 metabolites from both timepoints were used. A set of 42 metabolite ratios and 48 sums linked to specific enzymatic or other biological functions previously reported [25] were calculated and was added to the dataset (Supplementary Material 1). All metabolite variables were log2-transformed and scaled separately per timepoint.

### Statistical analysis

Associations between DP, food groups and nutrients with individual metabolite levels were analysed by linear mixed models (LMM), adjusting for sex, energy intake (kcal/day), body mass index (z-score) [26], season and age (visit), while subject and country (Germany, Belgium, Italy, Poland, or Spain) were included as random effects. All numerical covariates were scaled for the analysis. To control for LMM multiple testing,* p*-values were adjusted by the Benjamini-Hochberg false discovery rate (FDR) correction (q < 0.05). Ratio and sum associations were considered informative beyond the individual metabolite associations only if the* p*-value for the ratio or sum was lower than the smallest* p*-value observed for the single metabolites (*p-gain* = min (*p*-values of single metabolites contained in ratio (or sum))/* p*-value (ratio (or sum)) ≥ 1) [27]. Sensitivity analysis was conducted by fitting separate LMMs to the 5.5-year and 8-year data, excluding subject as random effect and age as fixed effect in the models. Differences in beta estimates were tested using Wald z-tests. In addition, Pearson correlation between beta estimates of the two timepoints was calculated.

The relationship between DP and groups of correlated metabolites was examined using metabolite set enrichment analysis (MSEA). Metabolites were first grouped via hierarchical clustering based on their Pearson correlation matrix. To perform clustering, a dissimilarity matrix was derived as 1 − correlation, and hierarchical clustering was conducted using complete linkage. The dendrogram was visually inspected to select a cut height that balanced cluster size with biological plausibility. A cut height of 0.8 resulted in 39 metabolite groups, of which 23 contained at least three metabolites and were included in the MSEA. Using the assignments of metabolites to these groups to define metabolite sets and the metabolite β estimates from the LMM with DP described above, the MSEA was performed applying the *fgsea* R package (version 1.30.0) the MSEA [28] with 1000 permutations. The analysis yielded a normalized enrichment score (NES) for each metabolite group, indicating the enrichment of metabolites in the group in the association results. A high and positive NES indicates that metabolites from this group are enriched (e.g., concentrated) at the top of the metabolite list ranked by β estimate, suggesting they are more abundant in individuals with stronger adherence to the DP. Conversely, a negative NES indicates that metabolites of the respective group are enriched in the associations with negative effect directions, suggesting that these metabolites tend to be less abundant with stronger adherence to the DP. NES values were adjusted by group size to account for differences in the metabolite group composition. Statistical significance of a NES was determined using a permutation-based approach. P-values were adjusted for multiple-testing with Benjamini-Hochberg. All statistical analyses were performed using R (version 4.4.0).

### Ethics

The study was conducted in accordance with the Declaration of Helsinki [29] and the standards of good clinical practice [30]. Ethics committees at each study centre approved the BiomarKid study protocol (IISPV 095/2022, LMU Ethik-Kommission-Nr: 02070 & Project Nr: 555 − 15 UMIL 0009763, CHC Liege 13/48/671, CHMI n°682).

## Results

### Participant’s characteristics

We included 421 children at ages 5.5 and 8 years from the EU CHOP study with dietary intake and metabolite data available. Of these, 156 children had data from both timepoints. Main characteristics are presented in Table 1.

**Table 1:** Sample population characteristics

	Timepoint 5.5 years (± 1 month)	Timepoint 8 years (± 1 month)
Sample *n*	294	283
Age (years)	5.51 [5.49, 5.54]	8.03 [7.99, 8.07]
Sex n (%) girls	153 (52.0)	142 (50.2)
Fasting n (%)	275 (94.2)	262 (93.2)
Country n (%)		
Germany	16 (5.4)	19 (6.7)
Belgium	35 (11.9)	24 (8.5)
Italy	118 (40.1)	60 (21.2)
Poland	50 (17)	74 (26.1)
Spain	75 (25.5)	106 (37.5)
Season n (%)		
Winter	66 (22.6)	88 (31.2)
Spring	74 (25.3)	83 (29.4)
Summer	66 (22.6)	45 (16)
Fall	86 (29.5)	66 (23.4)
BMI (kg/m^2^)	15.64 [14.72, 16.82] ^#^	16.55 [15.06, 18.2]
BMI (z-score)	0.24 [− 0.38, 0.96] ^#^	0.44 [− 0.43, 1.29]
Energy intake (kcal/day)	1396 [1227, 1568] ^*^	1617 [1420, 1769]
Protein (g/day)	50.93 [42.52, 58.78] ^*^	59.99 [51.08, 71.29]
Fat (g/day)	53.46 [44.25, 62.18] ^*^	65.29 [54.29, 75.91]
Carbohydrates (g/day)	174.94 [148.71, 204.01] ^*^	192.11 [167.18, 221.63]
Dietary fibre (g/day)	9.64 [7.34, 11.69] ^*^	11.72 [9.52, 13.99]
CORE (z-score)	0.51 [− 0.05, 1.14] ^*^	0.89 [0.20, 1.66]
PROT (z-score)	0.02 [− 0.53, 0.49] ^*^	0.32 [− 0.30, 0.93]
F&S (z-score)	− 0.12 [− 0.55, 0.68] ^*^	− 0.03 [− 0.64, 1.02]

Age: at blood collection; BMI: body mass index; CORE: core foods dietary pattern; PROT: animal protein sources dietary pattern; F&S: poor quality fats and sugars dietary pattern; ^#^data was collected at 6 years; ^*^data was collected at 5 years; numerical variables were expressed as median [25th percentile, 75th percentile]

### Metabolites associated with DP

A total of 48 out of the 236 metabolites quantified in 577 blood samples were significantly associated with at least one of the three DPs. Specifically, 23, 18, and 12 metabolites were associated with the CORE, PROT and F&S DP in the LMM, respectively (*p* < 0.05) (Table 2). Although individual metabolite associations did not generally remain significant after FDR correction, several associations showed consistent biological patterns across multiple related metabolites, metabolite ratios, and enrichment analyses.

Among the 23 metabolites associated with CORE, 12 metabolites were phosphatidylcholines (PCs), many of which contained docosahexaenoic acid (DHA, C22:6) as one of the two fatty acid sidechains [31]. PCs with polyunsaturated fatty acids (PUFAs), such as PC aa C38:6, PC ae C38:6 and PC ae C40:6, were higher in children with greater CORE adherence.

**Table 2:** Metabolites associated with DP

	CORE			PROT			F&S				CORE			PROT			F&S		
	β	*p*	q	β	*p*	q	β	*p*	q		β	*p*	q	β	*p*	q	β	*p*	q
Alanine	− 0.110	**0.030**	0.504	0.000	0.993	0.998	0.188	**0.002**	0.211	PC aa C40:4	− 0.103	**0.037**	0.533	− 0.065	0.192	0.745	− 0.028	0.554	0.917
Glycine	0.008	0.874	0.981	− 0.020	0.700	0.951	0.132	**0.028**	0.497	PC aa C42:1	0.000	0.997	0.999	0.039	0.475	0.896	− 0.161	**0.027**	0.493
Tyrosine	− 0.098	**0.049**	0.566	− 0.004	0.940	0.991	0.038	0.527	0.908	PC aa C43:6	0.003	0.958	0.993	0.112	**0.032**	0.512	0.036	0.564	0.920
α-Aminoadipic acid	− 0.048	0.326	0.838	0.073	0.171	0.734	0.089	**0.050**	0.566	PC aa C44:12	0.052	0.283	0.811	0.095	**0.035**	0.522	0.025	0.630	0.935
Taurine	0.102	**0.037**	0.533	0.055	0.268	0.802	− 0.021	0.724	0.958	PC ae C26:1	− 0.059	0.245	0.783	− 0.102	**0.042**	0.549	0.071	0.193	0.746
FFA 15:1	0.026	0.618	0.933	− 0.021	0.688	0.949	− 0.160	**0.013**	0.408	PC ae C34:0	0.070	0.184	0.740	− 0.105	**0.046**	0.557	− 0.159	**0.014**	0.410
FFA 18:0	0.099	**0.050**	0.566	0.014	0.789	0.969	− 0.058	0.357	0.851	PC ae C34:2	0.103	**0.043**	0.550	0.090	0.077	0.619	0.072	0.251	0.790
FFA 20:2	− 0.018	0.714	0.955	0.111	**0.027**	0.494	− 0.018	0.769	0.966	PC ae C34:3	0.025	0.626	0.934	0.155	**0.003**	0.216	0.114	0.070	0.611
FFA 20:5	0.120	**0.021**	0.468	0.040	0.445	0.888	− 0.054	0.400	0.871	PC ae C34:4	0.102	**0.029**	0.500	− 0.018	0.696	0.951	− 0.013	0.760	0.965
FFA 26:3	0.043	0.280	0.808	0.114	**0.004**	0.272	− 0.005	0.916	0.987	PC ae C36:4	0.052	0.313	0.832	0.125	**0.014**	0.411	0.033	0.587	0.927
FFA 26:5	0.067	0.205	0.754	0.019	0.720	0.956	− 0.140	**0.025**	0.483	PC ae C36:5	0.040	0.439	0.884	0.109	**0.034**	0.517	0.012	0.848	0.978
C10:2	− 0.065	0.233	0.775	− 0.066	0.235	0.775	0.161	**0.014**	0.410	PC ae C38:6	0.121	**0.023**	0.473	0.121	**0.021**	0.471	− 0.031	0.630	0.935
C16:0	− 0.085	0.117	0.681	− 0.145	**0.008**	0.340	− 0.010	0.879	0.983	PC ae C40:1	0.116	**0.017**	0.440	− 0.055	0.213	0.760	0.003	0.959	0.993
C9:0	− 0.014	0.795	0.971	− 0.137	**0.011**	0.404	0.075	0.253	0.791	PC ae C40:6	0.115	**0.028**	0.497	0.028	0.589	0.928	− 0.041	0.514	0.903
LPC a C18:2	0.032	0.533	0.909	0.066	0.195	0.746	0.129	**0.042**	0.549	SM C32:2	− 0.091	0.066	0.602	− 0.149	**0.003**	0.229	− 0.112	0.071	0.611
LPC a C22:6	0.108	**0.038**	0.540	0.009	0.855	0.978	− 0.140	**0.025**	0.484	SM C39:1	0.047	0.370	0.860	0.070	0.185	0.742	− 0.127	**0.035**	0.522
PC aa C20:0	− 0.015	0.748	0.962	− 0.099	**0.038**	0.540	0.049	0.216	0.763	SM C40:2	0.096	**0.032**	0.512	0.026	0.575	0.923	− 0.080	0.055	0.582
PC aa C30:0	− 0.150	**0.005**	0.281	− 0.122	**0.023**	0.473	− 0.058	0.379	0.864	SM C41:1	0.051	0.327	0.839	− 0.041	0.437	0.884	− 0.143	**0.023**	0.473
PC aa C32:1	− 0.079	0.104	0.662	− 0.111	**0.022**	0.473	− 0.051	0.402	0.872	SM C42:1	0.138	**0.010**	0.387	0.046	0.395	0.869	− 0.044	0.505	0.901
PC aa C32:3	0.026	0.617	0.933	− 0.109	**0.036**	0.530	− 0.041	0.512	0.902	SM C42:2	0.124	**0.018**	0.449	0.027	0.602	0.931	− 0.033	0.596	0.930
PC aa C36:0	0.112	**0.042**	0.549	0.028	0.617	0.933	− 0.013	0.842	0.978	SM C42:3	0.112	**0.019**	0.454	0.007	0.884	0.983	0.015	0.781	0.969
PC aa C38:0	0.139	**0.008**	0.345	0.042	0.416	0.876	− 0.091	0.111	0.671	Unknown_660.5	− 0.147	**0.002**	0.180	− 0.015	0.782	0.969	0.041	0.491	0.899
PC aa C38:3	− 0.101	**0.043**	0.549	− 0.062	0.221	0.767	− 0.077	0.164	0.728	Unknown_684.5	− 0.006	0.903	0.985	− 0.113	**0.030**	0.503	− 0.107	0.086	0.633
PC aa C38:6	0.135	**0.012**	0.408	− 0.003	0.949	0.992	− 0.092	0.159	0.725	Unknown_874.7	0.112	**0.029**	0.498	− 0.001	0.988	0.998	0.056	0.374	0.862

Values in bold are statistically significant (p or q ≤ 0.05). CORE: core foods dietary pattern; PROT: animal protein sources dietary pattern; F&S: poor quality fats and sugars dietary pattern; β: estimated regression coefficient from the linear mixed model; p:* p*-value; q: false discovery rate adjusted p-value; FFA: free fatty acid; C10:2 decadienoylcarnitine; C16:0 palmitoylcarnitine; C9:0 nonanoylcarnitine; LPC a: lysophosphatidylcholine (acyl); PC aa: phosphatidylcholine diacyl; PC ae: phosphatidylcholine acyl-alkyl; SM: sphingomyelin

In the case of PROT, most associated metabolites were also PCs, showing notable positive associations with several long-chain, polyunsaturated PCs (e.g., PC aa C43:6, PC aa C44:12, PC ae C36:5) and negative associations with shorter and saturated PCs (e.g., PC aa C20:0, PC aa C30:0, PC aa C32:1). The F&S pattern showed the lowest number of associated metabolites and the most diverse ones.

**Fig. 1:**
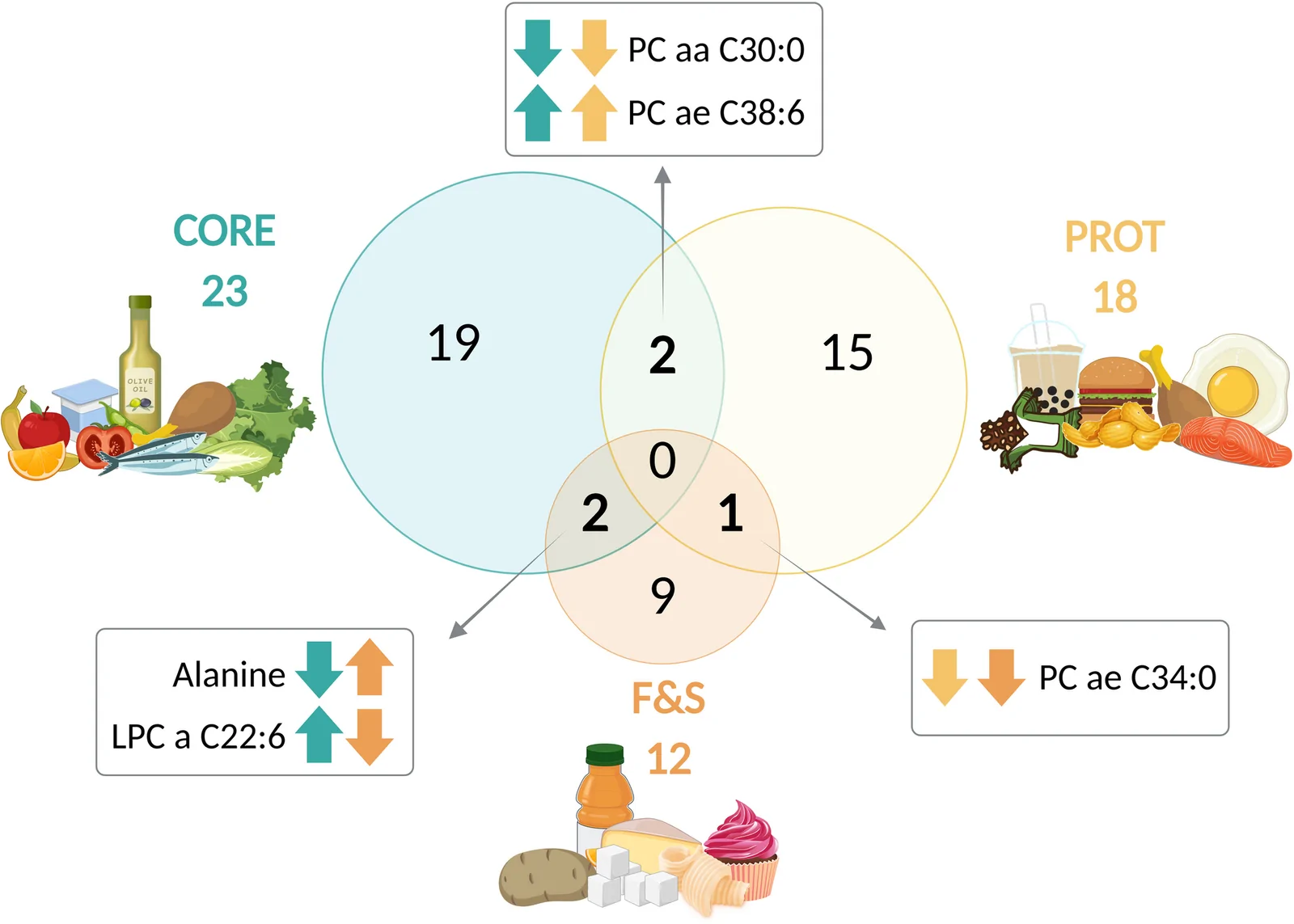
Overview association results between metabolites and dietary patterns (DPs). In total, 48 metabolites were associated with at least one DP. Five metabolites were associated with more than one DP. CORE: core foods dietary pattern; PROT: animal protein sources dietary pattern; F&S: poor quality fats and sugars dietary pattern; LPC a: lysophosphatidylcholine (acyl); PC aa: phosphatidylcholine diacyl; PC ae: phosphatidylcholine acyl-alkyl

Five metabolites were associated with more than one DP (Fig. 1). CORE and PROT were inversely associated with PC aa C30:0 and directly associated with PC ae C38:6. Opposite effect directions were observed for alanine and LPC a C22:6 with the CORE and F&S. Higher adherence to PROT and F&S were associated with lower levels of PC ae C34:0.

Associations between DPs and metabolite sums and ratios are presented in Table 3. After FDR correction, only the ratio of PC aa C40:5/PC aa C40:6 was significantly associated with CORE (p-gain: 3188.84). In contrast, this ratio was positively associated with F&S (p-gain: 27.94). The Fischer ratio (branched-chain AAs/aromatic AAs) was positively associated with CORE (p-gain: 10.39) and inversely with F&S (p-gain: 23.75). The ratio of long-chain FFAs/very long chain FFAs was inversely associated with PROT (p-gain: 1.81) and F&S (p-gain: 2.21). Ratio of PC aa C38:3/PC aa C38:4 was negatively associated with CORE (p-gain: 1.08), PROT (p-gain: 56.02) and F&S (p-gain: 4.01).

**Table 3:** Sums and ratios associated with DP

	CORE			PROT			F&S		
	β	*p*	q	β	*p*	q	β	*p*	q
Sums									
Polyunsaturated acylcarnitines	-0.072	0.176	0.738	− 0.084	0.116	0.680	0.145	**0.024**	0.479
Ratios									
Fischer ratio	0.118	**0.009**	0.380	0.030	0.551	0.915	− 0.106	**0.013**	0.409
Long chain FFAs/Very long chain FFAs	− 0.021	0.659	0.942	− 0.095	**0.045**	0.553	− 0.115	**0.050**	0.566
C2/C0	0.107	**0.042**	0.549	0.024	0.643	0.937	− 0.077	0.231	0.774
C2 + C3/C0	0.108	**0.040**	0.542	0.023	0.665	0.944	− 0.077	0.229	0.772
C16 + C18/C0	0.025	0.646	0.938	− 0.152	**0.005**	0.281	− 0.052	0.432	0.881
C0/Acylcarnitine C16:0	0.026	0.633	0.936	0.170	**0.002**	0.180	0.059	0.372	0.861
Acylcarnitine C8:0/Acylcarnitine C16:0	− 0.003	0.947	0.992	0.110	**0.028**	0.495	0.116	0.062	0.596
Monounsaturated PCs/Saturated PCs	− 0.108	**0.039**	0.541	0.018	0.736	0.960	0.067	0.279	0.808
Polyunsaturated PCs/Monounsaturated PCs	0.041	0.368	0.859	0.089	0.052	0.573	0.138	**0.015**	0.430
PC aa C36:3/PC aa C36:4	0.025	0.611	0.932	− 0.095	0.054	0.581	− 0.105	**0.016**	0.434
PC aa C38:3/PC aa C38:4	− 0.109	**0.039**	0.541	− 0.150	**0.004**	0.263	− 0.127	**0.041**	0.545
PC aa C40:5/PC aa C40:6	− 0.207	**3.82 × 10** ^**− 5**^	**0.016**	− 0.042	0.414	0.875	0.136	**0.020**	0.463

Table includes metabolite sums and ratios significant at least with one DP (*p* ≤ 0.05) and p-gain ≥ 1. Values in bold are statistically significant (p or q ≤ 0.05). CORE: core foods dietary pattern; PROT: animal protein sources dietary pattern; F&S: poor quality fats and sugars dietary pattern; β: estimated regression coefficient from the linear mixed model; p:* p*-value; q: false discovery rate adjusted* p*-value; Fischer ratio: branched-chain amino acids (AAs)/aromatic AAs; FFAs: free fatty acids; C2/C0: acetylcarnitine/free carnitine; C2 + C3/C0: acetyl- and propionyl-carnitines/free carnitine; C16 + C18/C0: palmitoylcarnitine + stearoylcarnitine/ free carnitine; C0/ C16:0: free carnitine/ palmitoylcarnitine; C8:0/C16:0: octanoylcarnitine/palmitoylcarnitine; PC: phosphatidylcholine; PC aa: phosphatidylcholine diacyl

### Metabolites associated with food groups and nutrient intake

PCs containing PUFAs, such as PC aa C38:6, PC ae C38:6, PC aa C40:6 and PC ae C40:6, were higher in children with greater fish intake (Table 4).

**Table 4:** Metabolites associated with food groups and nutrient intake

	Metabolite	β	p	q		Metabolite	β	p	q
Carbohydrates	PC aa C38:0	− 0.276	9.64 × 10^−5^	**0.030**	Animal protein	FFA 26:4	− 0.199	5.40 × 10^−5^	**0.021**
	PC ae C36:4	− 0.318	1.99 × 10^−5^	**0.011**		PC aa C30:0	− 0.174	1.17 × 10^−3^	0.147
	PC ae C36:5	− 0.292	1.07 × 10^−4^	**0.032**		PC aa C38:0	0.189	1.78 × 10^−4^	**0.046**
	PC ae C38:6	− 0.346	7.03 × 10^−6^	**0.005**		PC ae C34:2	0.221	1.51 × 10^−5^	**0.009**
	SM C41:1	− 0.284	1.85 × 10^−4^	**0.047**		PC ae C36:3	0.208	5.76 × 10^−5^	**0.021**
	SM C41:2	− 0.305	6.44 × 10^−5^	**0.021**		PC ae C36:4	0.301	5.30 × 10^−9^	**< 0.001**
Total sugar	Alanine	0.194	2.92 × 10^−4^	0.064		PC ae C36:5	0.309	2.30 × 10^−9^	**< 0.001**
	Fischer ratio	− 0.188	3.21 × 10^−4^	0.065		PC ae C38:5	0.230	6.78 × 10^−6^	**0.005**
	PC aa C30:0	0.163	3.09 × 10^−3^	0.232		PC ae C38:6	0.271	2.91 × 10^−7^	**0.001**
Grains	LPC a C16:0	0.210	5.76 × 10^−6^	**0.005**		PC ae C34:2/PC aa C32:2	0.232	1.09 × 10^−6^	**0.002**
	LPC a C16:1	0.193	2.27 × 10^−5^	**0.011**	Fish	PC aa C38:6	0.211	2.72 × 10^−6^	**0.004**
	LPC a C18:0	0.186	9.69 × 10^−5^	**0.030**		PC aa C40:6	0.221	5.11 × 10^−7^	**0.001**
	LPC/PC	0.225	6.43 × 10^−7^	**0.002**		PC ae C38:6	0.180	6.17 × 10^−5^	**0.021**
Healthy fats	Alanine	− 0.196	6.31 × 10^−4^	0.097		PC ae C40:0	0.208	3.77 × 10^−6^	**0.004**
	Fischer ratio	0.171	1.41 × 10^−4^	**0.040**		PC ae C40:6	0.171	1.05 × 10^−4^	**0.032**
	LPC a C22:6	0.231	6.30 × 10^−5^	**0.021**		PC aa C40:5/PC aa C40:6	− 0.278	1.00 × 10^−10^	**< 0.001**
	PC aa C38:6	0.256	2.13 × 10^−5^	**0.011**	Processed meat	Pyruvic acid	− 0.158	1.34 × 10^−4^	**0.038**
	PC ae C38:6	0.221	1.96 × 10^−4^	**0.048**		TCA cycle	− 0.173	3.03 × 10^−5^	**0.013**
	PC ae C40:6	0.220	1.57 × 10^−4^	**0.043**	Dairy protein	LPC e C16:0	0.192	2.64 × 10^−5^	**0.012**
	PC aa C40:5/PC aa C40:6	− 0.232	2.28 × 10^−5^	**0.011**		PC aa C30:0	0.159	7.44 × 10^−4^	0.108
Olive oil olives	PC aa C40:5/PC aa C40:6	− 0.202	1.71 × 10^−4^	**0.045**		Polyunsaturated FFAs/Saturated FFAs	− 0.169	1.96 × 10^−4^	**0.048**
Processed food fats	FFA 15:1	− 0.197	1.11 × 10^−4^	**0.032**		SM C32:1	0.170	5.54 × 10^−5^	**0.021**
Protein	PC ae C36:4	0.333	1.30 × 10^−6^	**0.002**		SM C39:1	0.188	5.74 × 10^−5^	**0.021**
	PC ae C36:5	0.338	9.49 × 10^−7^	**0.002**	Milk yogurt	PC aa C32:1	0.174	3.76 × 10^−5^	**0.016**
	PC ae C38:6	0.329	3.81 × 10^−6^	**0.004**		PC ae C34:0	0.201	1.49 × 10^−5^	**0.009**
	PC ae C34:2/PC aa C32:2	0.266	2.15 × 10^−5^	**0.011**		Polyunsaturated PCs/Monounsaturated PCs	− 0.150	1.48 × 10^−4^	**0.041**

Values in bold indicate q-values ≤ 0.05. β: estimated regression coefficient from the linear mixed model; p:* p*-value; q: false discovery rate adjusted p-value; PC aa: phosphatidylcholine diacyl; PC ae: phosphatidylcholine acyl-alkyl; SM: sphingomyelin; Fischer ratio: branched-chain AAs/aromatic AAs; LPC a: lysophosphatidylcholine (acyl); FFA: free fatty acid; TCA cycle: tricarboxylic acid cycle

Specifically, PC ae C38:6 was directly associated with total protein, animal protein, healthy fats, and indirectly associated with carbohydrates. PC ae C36:4 and PC ae C36:5 showed a similar pattern, as they were positively associated with protein and animal protein intake and negatively associated with carbohydrates. In contrast, PC aa C30:0 was indirectly associated with animal protein and directly associated with total sugar and dairy protein.

LPC a C22:6 was associated with healthy fats intake, and alanine was associated with both total sugar and healthy fats.

The ratio of PC aa C40:5/PC aa C40:6 was significantly associated with healthy fats (p-gain: 114.77), fish consumption (p-gain: 7160.98), and the olive oil and olives food group (p-gain: 938.30).

The Fischer ratio (branched-chain AAs/aromatic AAs) was positively associated with healthy fats, (p-gain: 854.90) and inversely with total sugar consumption (p-gain: 738.64).

There were positive associations between LPC a C16:0, LPC a C16:1, and LPC a C18:0 with the grain’s food group, which included both wholemeal and refined cereals. The LPCs/PCs ratio was also positively associated with grains, providing more information than each LPC alone (p-gain: 38.05).

PC aa C32:1 and PC ae C34:0 were positively associated with milk and yogurt food group, while the ratio of polyunsaturated PCs/monounsaturated PCs was negatively associated (p-gain: 143.34). LPC e C16:0 was positively associated with dairy protein, as were two SMs, SM C32:1 and SM C39:1. Conversely, the ratio of polyunsaturated FFAs/saturated FFAs was negatively associated with dairy protein (p-gain: 506.04).

Metabolites related to tricarboxylic acid cycle were negatively associated with processed meat (p-gain: 4.42), especially the pyruvic acid.

### Sensitivity analysis

Sensitivity analyses conducted separately at 5.5 and 8 years showed overall directional consistency of effect estimates across the two timepoints. Several key associations, including those involving fish-related phosphatidylcholines and the PC aa C40:5/PC aa C40:6 ratio, were observed at both ages. These findings suggest that the identified metabolomic patterns are not driven by a single measurement occasion and support the internal consistency of the results (Supplementary Material 2). All significant associations between metabolites, sums, and ratios with dietary data at each timepoint are presented in Supplementary Material 3. For instance, PC ae C40:6 was positively associated with fish consumption at 5.5 years, and a comparable trend was seen at 8 years, although it did not reach statistical significance. Similarly, the ratio PC aa 40:5/PC aa C40:6 was negatively associated with fish intake at both timepoints, and the stronger estimate was observed at 5.5 years as well (β=-0.459 vs. β=-0.168; *p* = 6.01 × 10^− 4^). There were also associations observed at only one timepoint, such as PC aa C30:0, which was indirectly associated with PROT exclusively at 8 years (β=-0.223, *p* = 0.002).

### Metabolite groups enriched in associations with DP

**Fig. 2:**
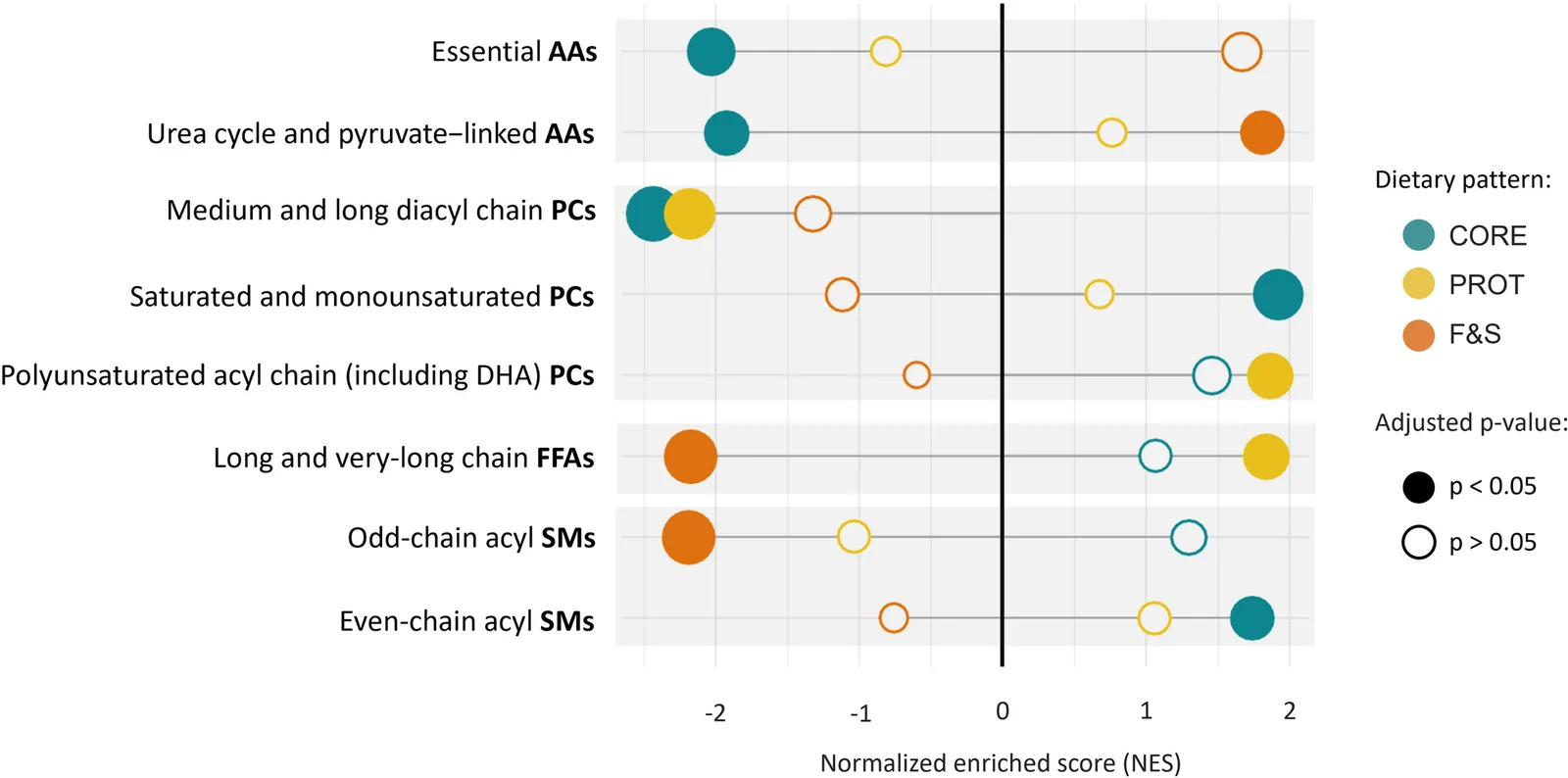
Metabolite set enrichment analysis of metabolite groups related to dietary patterns (DPs). CORE: core foods dietary pattern. PROT: animal protein sources dietary pattern. F&S: poor quality fats and sugars dietary pattern. The size of the circles represents the -log10-transformed p-values, with larger cycles indicating higher statistical significance. AAs: amino acids; PCs: phosphatidylcholines; FFAs: free fatty acids; SMs: sphingomyelins

Eight of the 23 metabolite groups included in MSEA (Supplementary Material 4) were significantly enriched for the associations with at least one DP (Fig. 2). CORE and F&S showed opposite directions in their NES for seven of the eight metabolite groups. In contrast, PROT did not follow such a consistent pattern.

A group of urea cycle and pyruvate-linked AAs (e.g., alanine, ornithine, threonine) showed a significant enrichment in inverse associations with CORE and a positive association with F&S. The same trends were seen with the essential AAs group (e.g., histidine, isoleucine, leucine), but the enrichment for positive associations with the F&S was not significant.

Both PROT and CORE were inversely associated with metabolites enriched for a group of medium and long diacyl chain PCs (e.g., PC aa C30:0, PC aa C36:1, PC aa C40:4). A group of PCs with high abundance of saturated and monounsaturated FAs (e.g., PC aa C36:0, PC aa C38:1, PC ae C40:1) and a group of even-chain acyl SMs (e.g., SM C34:1, SM C36:2, SM C42:1) were significantly enriched in positive associations with CORE.

A group of polyunsaturated PCs containing DHA (e.g., PC aa C38:6, PC aa C40:6, PC ae C40:6) and a group of long to very long chain FFAs (e.g., FFA 16:3, FFA 18:1, FFA 24:0) were enriched in positive associations with PROT. F&S was inversely associated with metabolites enriched for groups of long and very long chain FFAs (e.g., FFA 14:0, FFA 16:0, FFA 22:1) and odd-chain acyl SMs (e.g., SM C32:1, SM C33:1, SM C35:1).

## Discussion

This study provides novel insights into metabolomic signatures associated with DPs in childhood, using targeted metabolomics and a comprehensive approach to study dietary assessment intake. We identified specific metabolites and metabolite clusters associated with three distinct DPs in children aged 5.5 and 8 year, suggesting that metabolite profiles could reflect habitual dietary intake.

A key consideration when interpreting our findings is the high-dimensional nature of metabolomics data. As expected, most individual metabolite associations did not remain statistically significant after FDR correction. Therefore, our conclusions are not based on isolated metabolite findings but rather on the consistency of signals observed across several complementary analytical approaches, including individual metabolite analyses, metabolite ratios, metabolite set enrichment analyses, food-group analyses, and sensitivity analyses across two childhood timepoints. The convergence of evidence across these analytical levels increases confidence that the reported associations reflect biologically meaningful dietary signatures rather than random statistical fluctuations.

Our findings reveal interesting associations between DHA-containing PCs and a CORE foods DP (a dietary pattern considered healthy and characterised by fish intake, among others). Importantly, these results are consistent with previous report [13], further supporting their robustness. For instance, PC ae C38:6 (associated with CORE) has been previously associated with fish intake and negatively with sweets and bakery products [13]. The main dietary sources of PCs are choline-rich foods, such as eggs, meat and fish [32], as well as long-chain PUFAs containing DHA, which are predominantly found in oily fish [33, 34]. Notably, PC ae C38:6, together with PC aa C38:6 and PC ae C40:6, was associated both with fish consumption and with DP that included fish, reinforcing its value as a DP biomarker. In the MSEA, the metabolite group of DHA-containing PCs was associated with PROT rather than CORE, suggesting that the overall enrichment signal was driven by the coordinated behaviour of multiple metabolites within the set, many of which were also strongly associated with animal protein intake (e.g., PC ae C36:4, PC ae C36:5, PC ae C38:5 and PC ae C38:6).

Importantly, several metabolites were not identified solely through dietary pattern analyses but were consistently associated with specific food groups and nutrients expected to characterize those dietary patterns. This convergence across different dietary exposure metrics supports the biological plausibility of the findings and reduces the likelihood that the observed associations are due to chance alone.

PCs play a critical role in regulating tissue functions and energy metabolism, and variations in the FA composition of PC sidechains can lead to significant functional differences in cellular processes [31]. For example, the docosapentaenoic acid (DPA)/DHA ratio at higher values is considered to reflect an inflammatory status and has been related with the risk of obesity and other noncommunicable diseases [35]. In our study, PC aa C40:5/PC aa C40:6, which presumably reflects DPA/DHA ratio, was negatively associated with CORE, fish intake and olive oil, usually considered cardioprotective [15, 17, 35, 36].

Interestingly, LPC a C22:6 exhibited contrasting associations across dietary patterns, being positively associated with CORE and negatively linked to F&S. This bidirectional behaviour suggests that LPC a C22:6 may be a particular informative biomarker, capable of differentiating adherence to high-quality versus poor quality dietary patterns. Consistent with our findings, a negative association between LPC a C22:6 and a dietary pattern characterized by high consumption of meat and fast food was observed in adults, although this association was identified in the discovery phase and was not replicated in the validation sample [37]. Notably, higher circulating concentrations of LPC a C22:6 have previously been associated with a lower risk of incident type 2 diabetes in the PREDIMED study, supporting the potential relevance of this metabolite as a marker of favourable metabolic health in adults [38]. Together, these observations highlight LPC a C22:6 as a potentially informative metabolite; however, further studies are needed to confirm its utility as a robust biomarker of dietary quality across populations and age groups.

Plasma alanine was positively associated with adherence to F&S and inversely associated with CORE, consistent with the MSEA and previous reports revealing trends to inverse relationships with fruit intake [13] and a Mediterranean DP [39]. The glucose–alanine cycle supports hepatic gluconeogenesis during fasting, and thus alanine levels reflect shifts in energy substrate utilization [40]. Therefore, the higher alanine concentrations observed with adherence to the F&S could indicate impaired glucose handling and greater reliance on gluconeogenesis, whereas the inverse association with the CORE may reflect better metabolic efficiency.

Taurine showed a trend for a positive association with CORE and with olive oil. While the underlying mechanisms remain unclear, taurine has previously been inversely associated with components of metabolic syndrome, suggesting a potential protective role through antioxidant, anti-inflammatory, and endothelial-protective effects [41, 42].

In our study, a higher LPCs/PCs ratio, previously suggested as a marker of enhanced phospholipase A_2_ (PLA_2_) activity and pro-inflammatory states [43], was found to be associated with grain intake. Previous studies have also reported that consumption of refined grains increases PLA_2_ activity, whereas their substitution with whole grains reduces it and may improve glycaemic status [43]. Given the low consumption and limited variability of whole grain intake in our cohort, they were included within the overall grains category, which was predominantly composed of refined grains [18]. Thus, our finding on higher LPCs/PCs ratio associated with greater grains intake aligns with the existing literature.

### Strengths and limitations

Strengths of our study include a well-characterised, multicentre European paediatric cohort and the consideration of several confounders, which enhances the validity of our results. Furthermore, the analyses across two childhood timepoints, examining both individual metabolites and metabolite groups consistently associated with diet, support the internal consistency of the observed associations, as evidenced by the sensitivity analyses. The high dimensionality of metabolomics data relative to the sample size represents an important limitation. Although multiple-testing correction was applied throughout the analyses, most individual metabolite associations did not survive FDR adjustment and should therefore be interpreted as exploratory. Nevertheless, confidence in the findings is supported by several observations: (i) consistency across two childhood timepoints, (ii) concordant findings across individual metabolites, metabolite ratios, and metabolite set enrichment analyses, and (iii) biological coherence with known dietary sources and previously reported biomarkers. Independent replication in external cohorts remains necessary before these associations can be considered definitive biomarkers of dietary patterns.

Generally, we could corroborate that associations between metabolites and DPs were also consistently observed at the level of food groups and nutrients characterising those DPs. This multi-layered consistency strengthens the interpretation of our findings. Identifying associations across these different levels of dietary interpretation is key to uncovering robust and biologically meaningful relationships.

Adherence scores at ages 5.5 and 8 years were based on dietary patterns extracted at 2 years. While this approach ensured comparability of DPs scores across childhood, it may not have fully captured age-related changes in dietary behaviours and pattern composition. However, this strategy was chosen to enable longitudinal assessment of adherence to stable dietary patterns previously identified within the CHOP cohort, rather than deriving age-specific patterns that would not be directly comparable across timepoints. Previous analyses demonstrated that these dietary patterns remained largely consistent from 2 to 8 years of age and were associated with subsequent cardiometabolic outcomes.

The use of targeted metabolomics and measurements obtained at two timepoints across different analytical batches could have led to the exclusion of metabolites that could serve as potential biomarkers for diet and DP, thus overlooking certain aspects of metabolism. Although sample collection was standardized, some samples were not obtained under fasting conditions; this potential confounding effect was addressed by adjusting for country in the statistical analyses, as all non-fasting samples were from Germany. Nonetheless, biases in the interpretation of results cannot be ruled out.

## Conclusion

Distinct metabolites and metabolite groups were associated with adherence to specific dietary patterns in children, reflecting underlying nutritional exposures. The opposing metabolic signatures observed between the “Core foods” and “Poor-quality fats and sugars” patterns suggest that metabolomics can sensitively differentiate between health-promoting and adverse dietary behaviours. These findings provide exploratory evidence that metabolomic profiling may help characterize adherence to dietary patterns during childhood. The consistency observed across metabolite classes, enrichment analyses, and dietary components suggests biologically meaningful signatures. However, independent validation is required before these metabolites can be considered robust biomarkers of dietary patterns. Further research should aim to validate these findings and evaluate the identified metabolites as potential biomarkers of diet and health outcomes in other paediatric cohorts.

## Supplementary Information

Below is the link to the electronic supplementary material.

Supplementary Material 1

Supplementary Material 2

Supplementary Material 3

Supplementary Material 4

## Data Availability

The data that support the findings of this study are not publicly available due to access restrictions. Access to the data may be granted upon reasonable request and with permission from the data owner.

## Abbreviations

BiomarKid: Biomarker signatures of diet, physical activity and sleep in children and youth
DP: Dietary pattern
CORE: Core foods dietary pattern
PROT: Animal protein sources dietary pattern
F&S: Poor-quality fats and sugars dietary pattern
MSEA: Metabolite set enrichment analyses
LPC a: Lysophosphatidylcholine (acyl)
β: Estimated regression coefficient from the linear mixed model
p: *p*-value
PC aa: Diacyl-phosphatidylcholine
q: Adjusted* p*-values by Benjamini-Hochberg false discovery rate
DHA: Docosahexaenoic acid
NES: Normalized enrichment score
EU CHOP: EU Childhood Obesity Project
EDTA: Ethylenediaminetetraacetic acid
MS: Mass spectrometry
QC: Quality control
CP: Control plasma, CP-1 and CP-II
FIA–MS/MS: Flow-injection analysis–mass spectrometry
LMM: Linear mixed models
FDR: False discovery rate
BMI: Body mass index
PC: Phosphatidylcholine
PUFA: Polyunsaturated fatty acid
FFA: Free fatty acid
C10:2: Decadienoylcarnitine
C16:0: Palmitoylcarnitine
C9:0: Nonanoylcarnitine
PC ae: Acyl-alkyl-phosphatidylcholine
SM: Sphingomyelin
Fischer ratio: Branched-chain AAs / aromatic AAs
AA: Amino acid
C2/C0: Acetylcarnitine / free carnitine
C2 + C3/C0: Acetyl- and propionyl-carnitines / free carnitine
C16 + C18/C0: Palmitoylcarnitine + stearoylcarnitine/ free carnitine
C0/C16:0: Free carnitine / palmitoylcarnitine
C8:0/C16:0: Octanoylcarnitine / palmitoylcarnitine
TCA cycle: Tricarboxylic acid cycle
DPA: Docosapentaenoic acid
PLA₂: Phospholipase A₂
