# Supplementary material for: Association of three different dietary patterns with the metabolomic profiles of children: the BiomarKid project
Source: Eur J Nutr. 2026 Sep 12;65(7):251. doi: 10.1007/s00394-026-04098-1 (PMC13570914; doi:10.1007/s00394-026-04098-1)
Supplement: Supplementary file 1 [file 394_2026_4098_MOESM2_ESM.pdf]

Supplementary material to:

ASSOCIATION OF THREE DIFFERENT DIETARY PATTERNS WITH THE METABOLOMIC PROFILES OF CHILDREN: THE BIOMARKID PROJECT

Materials and methods:

Metabolite profiling – annotation, labelling and quantification

Amino acids (AAs), organic acids, ketoacids, non-esterified fatty acids, homocysteine and cysteine were measured by high performance liquid chromatography electrospray ionisation QTRAP mass spectrometry (HPLC-ESI-QTRAP-MS/MS), whereas acylcarnitines, phosphatidylcholines (PCs) (PC aa: diacyl-phosphatidylcholine; PC ae: acyl-alkyl-phosphatidylcholine), lysophosphatidylcholines (LPCs) (LPC a: lysophosphatidylcholine (acyl); LPC e: lysophosphatidylcholine (alkyl)) and sphingomyelins (SMs) were analysed using flow-injection analysis–mass spectrometry (FIA–MS/MS). The multiple reaction monitoring (MRM)-based quantification performed via FIA–MS/MS does not allow the exact determination of bond type (a/e), distribution of carbon atoms in the two fatty acid residues (for PCs, SMs), and position of double bonds. Annotation of PCs, LPCs, and SMs follows a predefined systematic based on precursor masses assuming higher abundance of fatty acids with even-chain fatty acids. Five metabolites are provided without annotation, labelled as unknowns. Metabolite concentrations were calculated from the measured MS/MS signal intensities using stable isotope-labelled internal standards added during plasma protein precipitation. Calibration curves generated from standards of known concentration were used to relate signal intensity to absolute metabolite concentration. Sample signals were normalized to their corresponding internal standard and corrected for the dilution factor from the protein precipitation step, obtaining absolute metabolite concentrations in mmol/L.

Metabolite profiling - quality control

All quality control steps were performed separately per timepoint for all timepoints with metabolomics data available (5.5, 8, 11 years). First, clear sample outliers were removed either based on visual inspection of PCA and high missingness for study samples, or inspection of

distribution plots per metabolite for QC samples. Subsequently, extreme values of QC samples were removed for each metabolite by applying mean ± 3×SD as a threshold. QC samples were then used to account for technical batch effects. The value of each sample was divided by the mean of QC sample values for each metabolite within a batch and then multiplied by the mean of QC values of this metabolite over all batches. For batches with no available metabolite value for QC samples, batch correction was performed with the same equation as described above, but replacing QC samples with study samples. Following batch correction, CP-II extreme values outside the median ± 1.5×IQR were removed. Metabolites with coefficient of variation >30% in CP-II (or QC samples for FIA–MS/MS-measured metabolites), a dispersion ratio (SD of QC samples / SD of study samples) >50%, and missingness > 30% were excluded. Two samples that were not measured across all analytical platforms at 5.5 years were excluded to ensure consistency in the dataset. To account for differences between timepoints, CP-II samples measured consistently across timepoints were used. For each metabolite, values were divided by the mean CP-II level at the corresponding timepoint and multiplied by the overall mean CP-II across the three timepoints (5.5, 8, and 11 years). This correction could not be applied to FIA–MS/MS–measured metabolites as no CP-II measurements were provided.

Results

Sensitivity analysis

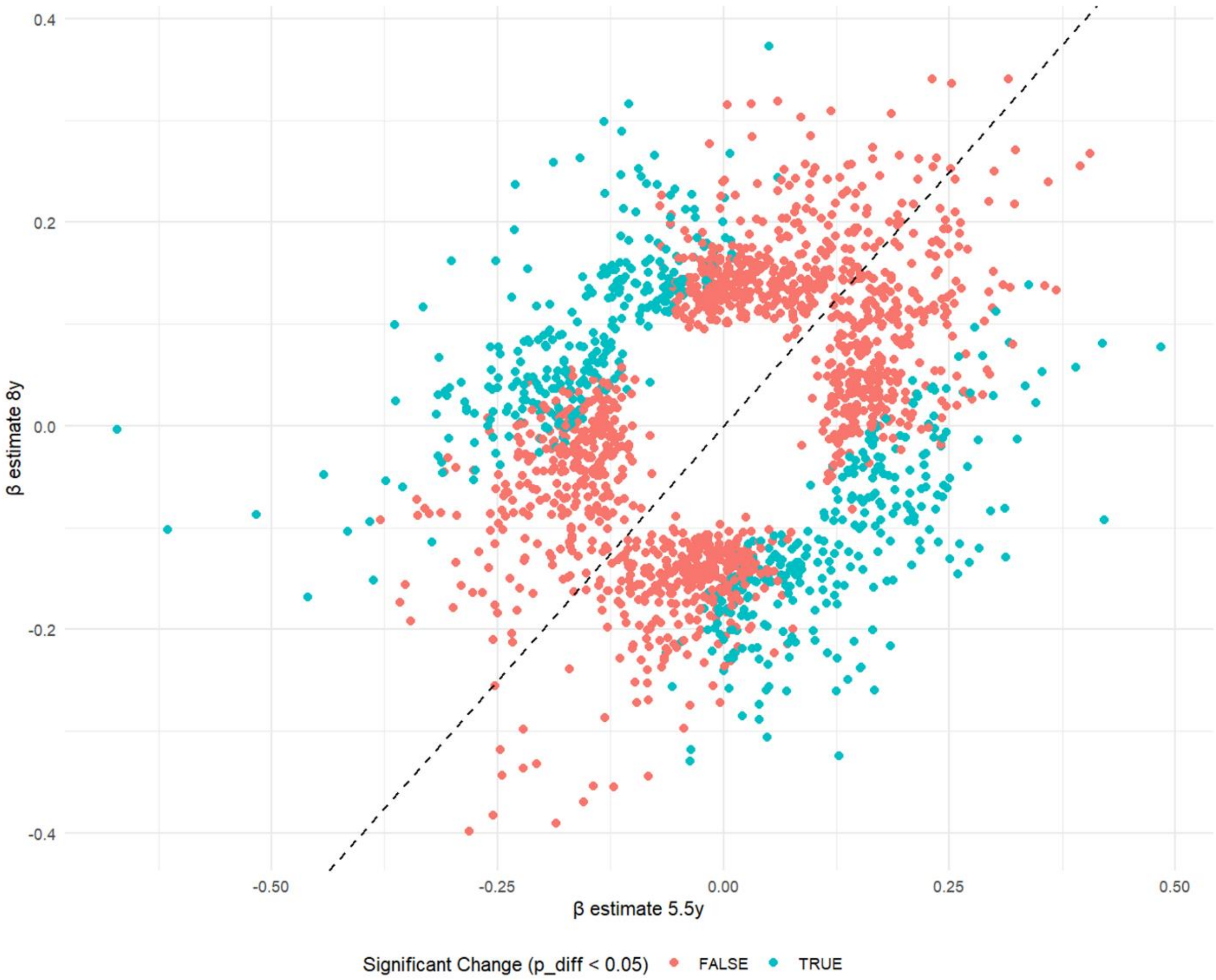

Supplementary figure 1. Scatter plot of significant β estimates across timepoints (sensitivity analysis). β estimates from linear mixed models assessing associations between metabolites and dietary patterns, food groups and nutrients at 5.5 and 8 years are shown. Points are coloured according to whether the differences in β estimates between timepoints were significant ($p_{diff} < 0.05$). Only associations that were significant at least at one timepoint are displayed.
